# Success Rate and Time for Bypassing the Fractured Segments of Four NiTi Rotary Instruments 

**DOI:** 10.22037/iej.v12i3.16866

**Published:** 2017

**Authors:** Alireza Adl, Arash Shahravan, Melika Farshad, Shahab Honar

**Affiliations:** a *Department of Endodontics, Biomaterials Research Center, School of Dentistry, Shiraz University of Medical Sciences, Shiraz, Iran;*; b *Endodontology Research Center, Kerman University of Medical Sciences, Kerman, Iran; *; c *Oral and Dental Disease Research Center, School of Dentistry, Shiraz University of Medical sciences, Shiraz, Iran; *; d *Postgraduate Student, Department of Periodontics, School of Dentistry, Shiraz University of Medical Sciences, Shiraz, Iran*

**Keywords:** Instrument Fracture, Instrument Separation, File Fracture, Fractured Instrument, NiTi Rotary File, Root Canal Treatment

## Abstract

**Introduction:**

The aim of this *in vitro* study was to compare the success rate and time required for bypassing the fractured segments of four different nickel-titanium (NiTi) rotary systems.

**Methods and Materials:**

This study was conducted on the mesiobuccal canals of 60 mandibular molars with fully-formed apices. Fifteen Flex Master, K3, RaCe and Hero Shaper instruments with 0.04 taper and tip size of #30 and 25 mm in length, were obtained. These instruments were notched at a point 3 mm from the tip of the instrument and were driven into the canals using a handpiece until the instruments fractured and became lodged therein. In the next step, an endodontist tried to bypass the fractured segment using K-files. The number of bypassed samples and the time required for bypassing of each sample were recorded. The *Chi*-square test was used to compare the bypassing rate among the experimental groups. One-way analysis of variance followed by Tukey’s post hoc test was conducted to compare the time taken for bypassing of the fractured fragments.

**Results:**

One instrument in Flex Master group and two broken segments in each of the K3 and Hero groups were not bypassed. All of the samples in RaCe group were bypassed. No significant difference was found among four tested groups regarding rate of bypassing (*P*=0.738). The time taken to bypass fragments in the Hero group was significantly more than in those of K3 (*P*=0.047) and RaCe (*P*=0.024).

**Conclusion:**

Under the limitations of this study, design features of rotary files can influence the time needed to bypass separated fragments.

## Introduction

Root canal debridement, cleaning and shaping are considered essential steps in root canal therapy. Endodontic hand and rotary files are the most commonly used instruments for removal of infected and affected dentin and for smoothing of the canal walls [1].

Advancements of the nickel-titanium (NiTi) alloy have led to development of practical files. Due to their high flexibility and superior resistance to torsional fracture, coupled with the design of cutting blades, NiTi engine-driven systems have become an important and common technique for root canal cleaning and shaping [2, 3].

Current experimental and clinical evidences show that the NiTi rotary system cleans out root canals, especially those with curvatures, far more smoothly and consistently with less chance of complications such as strip perforation, transportation and zipping compared to hand instruments [4, 5]. Moreover, faster treatment time of the rotary system can be a more comfortable and less frightening experience for patients [6].

However, the advent of the NiTi alloy has not resulted in a lower incidence of endodontic instrument fracture [7, 8] and NiTi files can be broken in the root canals without any significant evidence of damage on their surfaces [9-11]. The fracture rate of NiTi rotary instruments has been reported as being between 1.3% [12] and 10% [13]. The fracture of rotary instruments may affect the entire prognosis of root canal therapy [14]. 

In a study conducted by Spili *et al.* [15], the healing rates of teeth with periapical lesions were 87% for cases with a fractured instrument and 93% for matched controls after at least one year.

Conventional conservative management of separated instruments include attempts to remove the fragment, attempts to bypass the fragment, or preparing and filling the root canal system to the coronal level of the fragment [16, 17].

Removal of the fragment is considered as the optimal treatment option because cleaning and shaping of the root canal system can then be completed effectively to eliminate microorganisms. However, this treatment is a sophisticated process that needs training, experience and knowledge of the different methods, techniques and devices [18].

A major disadvantage of the retrieval of separated fragments has been excessive removal of root dentin coronal to the separated fragment, which may result in perforation or predispose the teeth to vertical root fracture [19-22].

Considering the potential complications of removal of separated instruments, bypassing a fragment may also be an appropriate treatment option. To some extent, this approach fulfills the objective of root canal treatment which are proper root canal preparation followed by good obturation [18].

Therefore, bypassing the separated instrument has been considered a successful approach [16, 23-26]. Moreover, in most cases, once bypassed, the fragment can be removed successfully [11, 27]. Many factors may influence the success of bypassing of separated rotary files in the root canals, including root canal anatomy, the location of fractured file segments and the design features of rotary files.

Currently there are many NiTi instrument systems on the market that are classified according to their design, shaping characteristics, number of flutes and clinical performance [28, 29]. There has been no previous study comparing the success rate of bypassing of the broken segments of different rotary files.

Therefore, the aim of this *in vitro* study was to compare the success rate and time required for bypassing the fractured segments of four different NiTi rotary systems: Flex Master (VDW, Munich, Germany); K3 (Syborn Endo, Orange, CA, USA); RaCe (FKG Dentaire, La-Chaux-de Fonds, Switzerland); and Hero Shaper (Micro-Mega, Besancon, France).

## Materials and Methods

Sixty human mandibular first molars extracted from patients of varying age for routine clinical reasons were used in this study. Teeth with completely formed root apices, two separate mesial canals, and an average length of 20±1 mm were included. Criteria for exclusion were: abnormal anatomies, double curvature, calcification, internal resorption, or crack. Root canals that could not be negotiated to the apical foramen with a #10 file or had a diameter exceeded #20 file were also excluded. Conventional access cavities were prepared and radiographs were taken. Only mesiobuccal canals which had a curvature less than 25 degrees were used.

In order to reduce the impact of canal anatomy on this study, the selected canals were enlarged up to file #20 using hand K-files (Mani, Tochigi, Japan), and the coronal part of the root canals was widened using #2 Gates Glidden drills (Mani, Tochigi, Japan).

**Table 1:** The number of bypassed and not bypassed samples in four experimental groups

**Rotary files**	**Bypassed**	**Not bypassed**
**Flex Master**	14	1
**K3**	13	2
**RaCe**	15	0
**Hero**	13	2

**Table 2:** The mean (SD), maximum and minimum of time (min) taken for bypassing of fractured segments

**Rotary files**	**Mean (SD)**	**Minimum**	**Maximum**
**Flex Master**	2.26 (1.87)^a,b^	0.5	6
**K3**	1.91 (1.48)^b^	0.5	5
**RaCe**	1.83 (1.38)^b^	0.4	4
**Hero**	4.03 (2.84)^a^	1	7

Different letters in the column indicate a statistically significant difference (P<0.05); same letters in the column indicate the differences are not significant (P>.05

**Figure 1.**
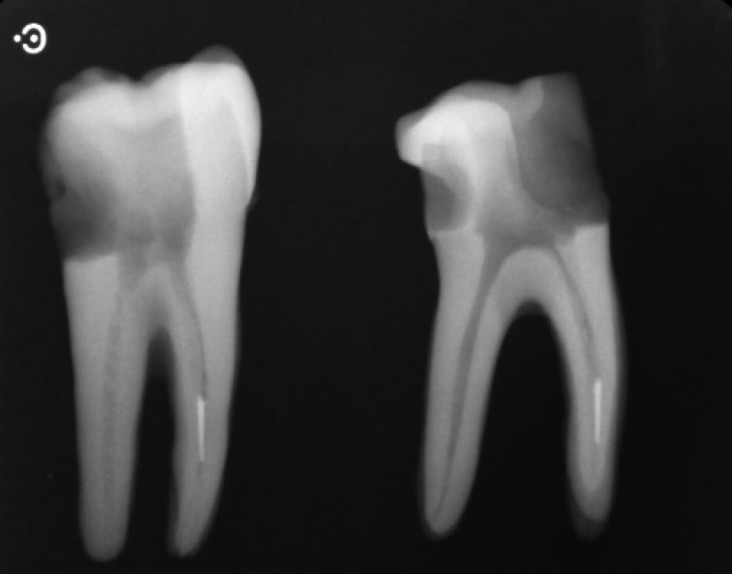
Samples after file fracture

The selected teeth were randomly divided in four groups of 15 teeth each. Fifteen instruments from four manufacturers including Flex Master (VDW, Munich, Germany), K3 (Syborn Endo, Orange, CA, USA), RaCe (FKG Dentaire, La-Chaux-de Fonds, Switzerland) and Hero Shaper (Micro-Mega, Besancon, France) with 0.04 taper, 25 mm length and ISO size of 30, were used in this study. According to a method suggested by Ward *et al.* [30], these instruments were notched to a depth of half the instrument thickness with a disk at a point 3 mm from the tip of the instrument. Then each group of notched instruments was driven into the respected mesiobuccal canals using a high-torque rotary headpiece and an electric motor with parameters set according to the manufacturer’s instructions, until the instrument fractured and became lodged therein. A light apical pressure was applied on the rotating instruments in order to control the location of instrument fracture. Parallel radiographs were taken to make sure that the fragments were fractured before the apical curvature (Figure 1). Cases in which the fragments were lodged beyond the curves were excluded and replaced. 

In the next step, an experienced endodontist who was blinded to the groups tried to bypass the fractured segment using K-files (Mani, Tochigi, Japan) of sizes 8, 10 and 15. During the bypassing procedure, the root canals were irrigated with 2.5% sodium hypochlorite (Golrang, Tehran, Iran). The time taken to bypass each sample was recorded from the moment the endodontist started bypassing until the moment that #15 file reached the working length of the root canal. At this step another parallel radiograph was obtained to make sure samples were bypassed and K-files were in the right path of the canal. Samples that were not bypassed after 30 min were considered non-bypassable. 

Chi-square test was used to compare the rate of bypassing among the experimental groups. One-way analysis of variance followed by Tukey’s post hoc test was conducted to compare the time taken for the bypassing of fractured fragments. All statistical analyses were performed at the 0.05 level of significance.

## Results

Five out of 60 samples were not bypassed after 30 min. Table 1 describes the number of bypassed samples for each group and Table 2 describes the mean (SD) and minimum and maximum time taken to bypass the file fragments in each of the four tested groups of samples. 

No significant difference was found among the four tested groups regarding rate of successful bypassing (*P*=0.738). There was a significant difference among the experimental groups in the time required for bypassing the fragments (*P*=0.020).

The time taken to bypass fragments in the Hero Shaper group was also significantly more than in those of the K3 (*P*=0.047) and RaCe (*P*=0.024) groups (Table 2).

## Discussion

The aim of this *in vitro *study was to evaluate the success rate and time required for bypassing fractured segments of four differently designed NiTi rotary files. 

NiTi rotary files are manufactured with several differences. These varieties include differences in the shapes of cross sections, number of flutes, rake angles, graduating tapers, *etc.* [31]. These design variations may also affect the ease of bypassing when they are fractured in the root canals. Instruments evaluated in this study were Flex Master, K3, RaCe and Hero Shaper. 

The instruments of the Flex Master system have a triangular convex cross-section without radial lands, and three cutting edges with a negative cutting angle. The instruments have a noncutting tip [1, 32]. Hero Shaper instruments have similar design features: a triangular cross-section with three cutting edges, a negative cutting angle and a noncutting tip [33, 34]. The K3 instruments have a slightly positive rake angle in combination with a so-called radial land relief and an asymmetrical cross-sectional design [35, 36]. The RaCe instruments possess a triangular cross-sectional design with alternating sharp cutting edges and a noncutting tip [1, 37].

The success rate of bypassing of the separated fragments in this study was very high, while in a clinical study only 37.5% of fractured files were successfully bypassed [26]. In another clinical study, the overall rate of success in retrieving or bypassing the fragments was 53%. Type of teeth, location of the fragment in the canal, degree of curvature, length of fragment, and type of fractured instrument were factors affecting the rate of success [25]. The high success rate of bypassing in the present study can be attributed to several factors related to the design of the study. In this study, the selected canals had minimal curvature and received an initial enlargement in order to reduce the impact of canal anatomy on the results. Using notched instruments may also have caused separation of the fragments without being tightly screwed into the canals. Moreover, the files were separated before the apical curvature.

Ward *et al.* [30] also demonstrated that the success rate of fractured segment removal significantly increased when the fractured segment was located coronal to the apical curvature. 

In this study, if the broken fragment could not be bypassed after 30 min, an unsuccessful bypass attempt was recorded. A 30-min time limit was used because it was judged that approximately 30 min would be the time available to attempt fractured-instrument bypass in a 60-min appointment. 

In this study, no significant difference was found among experimental groups regarding the bypass probability. However, this finding should be interpreted with caution because all samples in the RaCe group were successfully bypassed, while two samples in the K3 group and two samples in Hero group were not bypassed. Therefore, the lack of significant differences between the groups may be attributed to the small sample size of this study. Regarding the time taken to bypass the fragments, significant differences were detected among the groups. 

The Hero Shaper files were demonstrated to need the most time to be bypassed followed by Flex Master files. This finding can be credited to their similar design features. Both Hero and K3 files have a triangular cross-section with three cutting edges and a negative cutting angle.

In this study, RaCe was the only group in which all samples were successfully bypassed with significantly less time of bypassing compared with the Hero group. This may be attributed to the design characteristics of this file system. RaCe files are less twisted in a given length than other rotary files. Moreover, RaCe files have alternating cutting edges and this design is claimed to prevent the instrument from screwing into the root canal thus reducing intraoperative torque values.

One of the limitations of the current study was that files were fractured before the curvature of the root canals, while in a clinical situation, files may separate in or beyond the curvatures. Therefore, it is suggested that further investigations be undertaken to evaluate the effect of different file design on bypass probability of files that are fractured in different parts of canals.

## Conclusion

Within the limitations of this study, the design features of rotary files can influence the time needed to bypass the separated fragments located coronal to the canal curvature.
